# Alpha7 nicotinic acetylcholine receptor is required for amyloid pathology in brain endothelial cells induced by Glycoprotein 120, methamphetamine and nicotine

**DOI:** 10.1038/srep40467

**Published:** 2017-01-11

**Authors:** Liqun Liu, Jingyi Yu, Li Li, Bao Zhang, Lingjuan Liu, Chun-Hua Wu, Ambrose Jong, Ding-An Mao, Sheng-He Huang

**Affiliations:** 1 Department of Pediatrics, The Second Xiangya Hospital, Central South University, Changsha, Hunan, 410011, China; 2 Saban Research Institute, Childrens Hospital Los Angeles, University of Southern California, Los Angeles, CA90027, USA; 3 School of Public Health and Tropical Medicine, Southern Medical University, Guangzhou, 510515, China; 4 Department of Clinical Laboratory, Kunming Children’s Hospital, Kunming Medical University, Kunming, Yunnan 650034, China

## Abstract

One of the most challenging issues in HIV-associated neurocognitive disorders (HAND) caused by HIV-1 virotoxins and drug abuse is the lack of understanding the underlying mechanisms that are commonly associated with disorders of the blood-brain barrier (BBB), which mainly consists of brain microvascular endothelial cells (BMEC). Here, we hypothesized that Glycoprotein 120 (gp120), methamphetamine (METH) and nicotine (NT) can enhance amyloid-beta (Aβ) accumulation in BMEC through Alpha7 nicotinic acetylcholine receptor (α7 nAChR). Both *in vitro* (human BMEC) (HBMEC) and *in vivo* (mice) models of BBB were used to dissect the role of α7 nAChR in up-regulation of Aβ induced by gp120, METH and NT. Aβ release from and transport across HBMEC were significantly increased by these factors. Methyllycaconitine (MLA), an antagonist of α7 nAChR, could efficiently block these pathogenic effects. Furthermore, our animal data showed that these factors could significantly increase the levels of Aβ, Tau and Ubiquitin C-Terminal Hydrolase L1 (UCHL1) in mouse cerebrospinal fluid (CSF) and Aβ in the mouse brains. These pathogenicities were significantly reduced by MLA, suggesting that α7 nAChR may play an important role in neuropathology caused by gp120, METH and NT, which are the major pathogenic factors contributing to the pathogenesis of HAND.

Despite significant advances in highly active antiretroviral therapy (HAART), the prevalence of HIV-associated neurocognitive disorders (HAND) has significantly increased^1^. This is mainly due to the inability of antiretroviral drugs to cross the blood-brain barrier (BBB), the irreversible injury prior to starting therapy, HAART toxicity, and the role of CNS as the reservoir for HIV-1, which is capable of migrating out of the brain^1^,^2^,^3^. The incidence of acquired immune deficiency syndrome (AIDS) of central nervous is higher or accelerated among the aging populations and drug users^4^,^5^. The incidence of HAND also increases with age. Current projections suggest that more than 50% of HIV + patients in the United States are over 50 years old by 2015^4^. All these factors together, along with the fact that the entire HAND spectrum in therapeutic trials using neuroprotective or anti-inflammatory drugs for treatment of HAND is difficult to assess in clinical practice, exacerbate the problem of diagnosis/prognosis, prevention and treatment of these disorders^5^.

With advancing age, HIV + patients may potentially develop other neurodegenerative disorders, including Alzheimer’s disease^6^. The pathological hallmarks of Alzheimer’s disease are characterized by the development of amyloid-beta (Aβ) plaques and neurofibrillary tangles in the brain that ultimately result in neuronal death^7^. These Pathological changes take more 20 years before the onset of clinical symptoms of Alzheimer’s disease. The Aβ peptides, which are produced via the amyloidogenic pathway of amyloid precursor protein (APP) proteolysis, have been shown to be neurotoxic since they could mediate inflammation and oxidative stress^8^. An earlier study demonstrated that APP was accumulated in HIV encephalitis and that widespread axonal injury as well as an increased prevalence of amyloid plaques was found in brains of patients with AIDS compared with the age matched, non-HIV-infected controls^7^. This suggested that amyloid plaque formation could be facilitated by HIV-1-induced inflammatory response in the central nervous system (CNS)^6^. Since the firstly reported in 1997^7^, more studies have shown that amyloid deposition in the brains of HIV-1-infected patients was increased and correlated with the patient age^9^. There are cellular pathology similarities between Alzheimer’s disease and HAND^6^,^9^. Both animal and human studies demonstrated that Aβ metabolism could be altered by HIV-1 infection^6^,^9^. It has been suggested that the BBB may play a critical role in Aβ metabolism and homeostasis^9^.

One of the most challenging issues in HAND caused by HIV-1 virotoxins [e.g., Glycoprotein 120(gp120)] and related comorbid factors [e.g., methamphetamine (METH) and nicotine (NT)] is the lack of understanding the underlying mechanisms of the shared comorbidities that are commonly associated with disorders of the BBB, which mainly consists of brain microvascular endothelial cells (BMEC). Our recent study suggests that alpha7 nicotinic acetylcholine receptor (α7 nAChR) is an essential regulator of inflammation, which contributes to neuroinflammation and BBB disorders caused by microbial (e.g., HIV-1 Glycoprotein 41(gp41)/gp120, *Cryptococcus neoforman, E. coli*) and non-microbial (e.g., METH and nicotine) factors^10^,^11^,^12^. METH, NT and gp120 were able to significantly increase the blood levels of both molecular [Ubiquitin C-Terminal Hydrolase L1(UCHL1), S100B] and cellular (circulating BMEC, cBMEC) biomarkers for BBB injury. The enhancement of these biomarkers, CNS inflammation and BBB permeability was significantly reduced in α7 nAChR knockout mice, suggesting the involvement of this important inflammatory regulator in CNS disorders and BBB injury caused by microbial and non-microbial factors. Alpha7 nAChR also contributes to the pathogenesis of neurodegenerative disorders, including Alzeimer’s disease^13^. As mentioned above, Alzheimer’s-like brain pathology has been observed in patients with HIV-1 infection. HIV virotoxins could promote the secretion of Aβ (1–42) in primary rat fetal hippocampal cell cultures. However, the underlying mechanisms remain obscure and it is unclear whether the α7 nAChR cholinergic pathway is essential for the role of BBB disorders in the pathogenesis of HAND and Alzheimer’s-like brain pathology caused by multiple pathogenic factors. We hypothesized that gp120, METH and NT can enhance Aβ accumulation in BMEC through α7 nAChR-mediated signaling cascade, which is the common pathway.

## Results

### HIV-1 gp120, METH and NT exposure increases Aβ levels of the culture supernatants in human BMECs (HBMECs)

**Figure 1:**
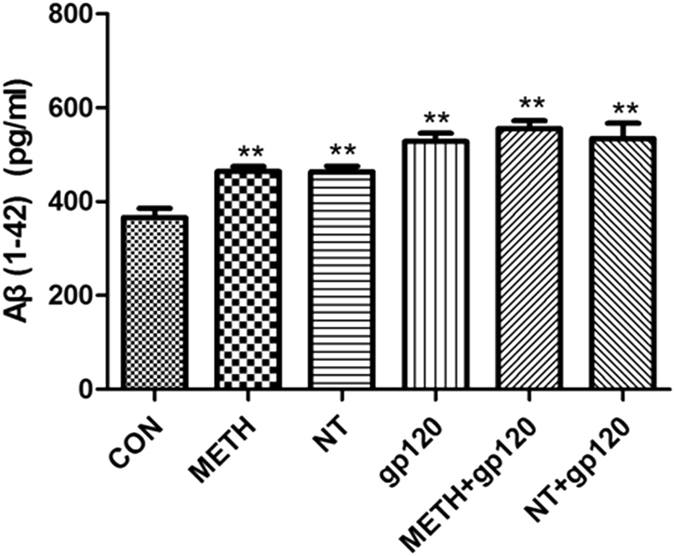
Aβ (1–42) accumulation in the culture supernatants (CS) from HBMECs exposed to HIV-1 gp120, NT and METH. The diagram shows results of direct anti-Aβ (1–42) ELISA measurements of the undiluted CS samples collected from HBMECs cultures (*n* = 6 per group) treated with gp120 (50 ng/ml), NT (10 μM) and METH (50 nM) either alone or in combination of them [METH (50 nM) + gp120 (50 ng/ml) or NT (10 μM) + gp120 (50 ng/ml)] for 24 h. The freshly prepared Aβ (1–42) stock was serially diluted with cell culture growth medium to generate standard ELISA calibration curves. Data were presented as mean values ± SEM. **Marks significant (*P* < 0.01) differences in extracellular Aβ (1–42) accumulation.

**Figure 2:**
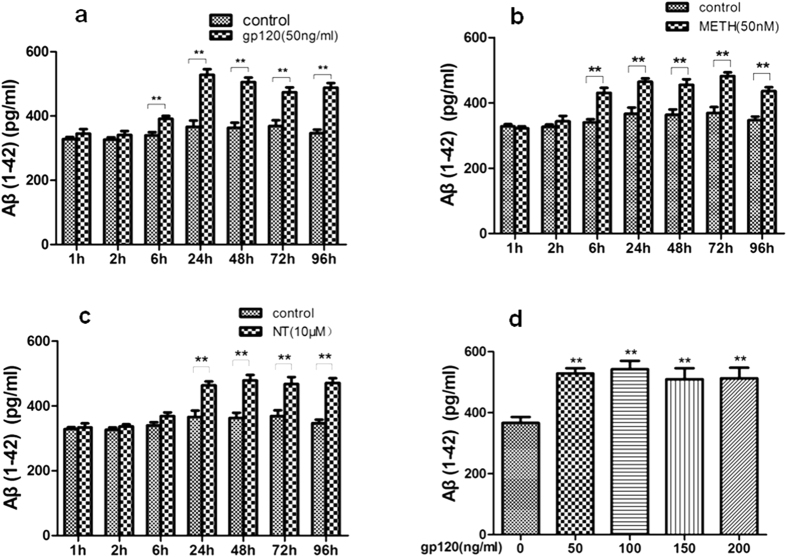
Dose- and time-dependent effects of HIV-1 gp120, METH and NT on the Aβ (1–42) release in HBMECs cultures. The Aβ (1–42) immunoreactivity was measured in CS samples collected from HBMECs cultures during the continuous exposure (1–96 h) to 50 ng/ml gp120 or vehicle (*n* = 6 per group). (**a**) 50 ng/ml gp120 (1–96 h); (**b**) 50 nM METH (1–96 h); (**c**) 10 μM NT (1–96 h); and (**d**) 0–200 ng/ml gp120 (24 h). Data are presented as mean values ± SEM. **Marks significant (*P* < 0.01) differences in Aβ (1–42) immunoreactivity between gp120- or METH/NT-treated and vehicle-treated cell cultures.

The blood-brain barrier (BBB) plays a critical role in regulating Aβ levels in the brain^14^. Therefore, we hypothesized that exposure to HIV-1 gp120, METH and NT might predispose brain endothelial cells to alterations of Aβ levels and thus contribute to brain amyloid pathology. To confirm the effects of HIV-1 gp120, NT and METH on cellular Aβ levels, HBMECs were treated with gp120 (50 ng/ml), NT (10 μM), and METH (50 nM) either alone or in combination of them [METH (50 nM) + gp120 (50 ng/ml) or NT (10 μM) + gp120 (50 ng/ml)] for 24 h. ELISA assays with the antibody specific to the 1–42 fragment of Aβ revealed the Aβ protein levels in HBMECs. As illustrated in Fig. 1, a 24 h exposure to HIV-1 gp120, NT or METH markedly increased Aβ expression in all experimental treatments as compared to the control cultures. In addition, HBMECs were exposed to different doses of gp120 (0–200 ng/ml) for 24 h or 50 ng/ml gp120, 50 nM METH, 10 μM NT at different time points (1–96 h), and subjected to ELISA assays. As indicated in Fig. 2, HIV-1 gp120 (Fig. 2a,d), METH (Fig. 2b) or NT (Fig. 2c) significantly increased the Aβ 1–42 levels in a dose- and time-dependent manner.

### Gp120-, METH- and NT-induced accumulation of Aβ *in vivo*/*in vitro* and gp120-induced Aβ transport is inhibited by MLA

**Figure 3:**
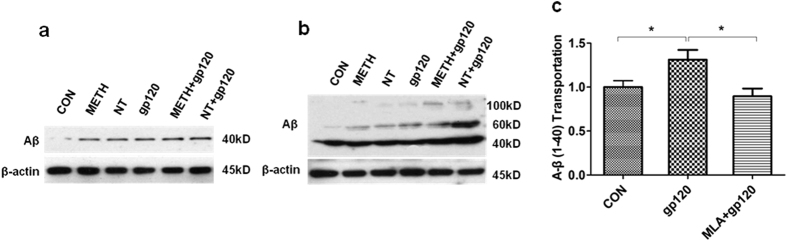
HIV-1 gp120, METH and NT exposure increased accumulation of Aβ *in vivo* and *vitro*. HBMECs were treated with HIV-1 gp120 (50 ng/ml), METH (50 nM), NT (10 μM), METH (50 nM) + gp120 (50 ng/ml) and NT (10 μM) + gp120 (50 ng/ml). C57BL/6 J mice were exposed to HIV-1 gp120 (50 ng/mouse, 2d), METH (2, 4, 6, 8, 10, 10, 10, 10, 10, 10 mg/kg/d), NT (1.5 mg/kg/d, 3d). After an exposure to HIV-1 gp120, METH, and NT, (**a** and **b**) Aβ accumulation in the culture supernatants from HBMECs (**a**) and whole mouse brain tissues (**b**) were made by Western blot analysis. (**c**) Inhibitory effects of the α7 antagonist (MLA) on gp120-induced Aβ (1–40) (Rhodamine green-labeled) release, transport across HBMECs changes in the cell cultures. Data were presented as mean values ± SEM. *Marks significant (*P* < 0.05), differences in Aβ (1–40) transport across HBMECs.

Aβ is a potential cause of Alzheimer’s disease, in which it accumulates in the brain and increases monocyte migration across the BBB. To confirm the effects of HIV-1 gp120, METH and NT on cellular Aβ levels *in vivo* and *vitro*. After an exposure to HIV-1 gp120, METH, and NT, HBMECs and mouse brain tissues were used for immunoblotting analysis. Immunoblot with the antibody specific to the 17–24 fragment of Aβ (SIG-39220 from Covance Research Products) revealed several Aβ-specific bands of molecular weights of ~40, 60, and 100 kDa in the brain tissues of C57BL/6 J mice (Fig. 3b). This antibody preferably reacts with the precursor and aggregated forms of Aβ proteins. These multiple Aβ immunoreactive bands may be caused by the formation of various Aβ oligomers as described earlier^15^, and/or by the binding of Aβ to other cellular proteins. Exposure to HIV-1 gp120, METH and NT significantly increased the Aβ levels in HBMECs and the brains of C57BL/6 J mice as compared to the controls (Fig. 3a,b). We also determined the effects of MLA on gp120-mediated elevation of Aβ transport. A 24 h exposure to gp120 results in a marked increase of Aβ transport. However, pretreatment with MLA appeared to decrease gp120-mediated increase in Aβ transport (Fig. 3c), suggesting that α7 nAChR plays an important role in Aβ homeostasis.

### Gp120- and METH-induced expression of biomarkers and receptors for BBB injury

**Figure 4:**
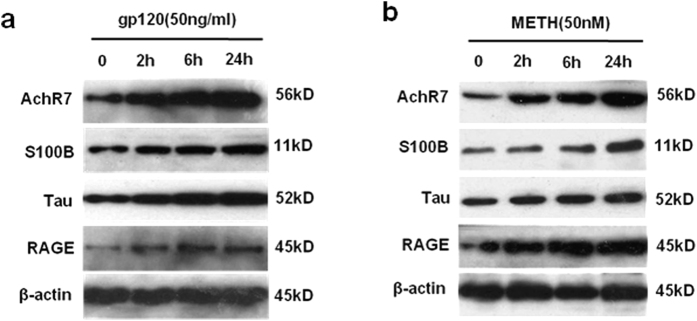
HIV-1 gp120- and METH-induced biomarkers accumulation in HBMECs. HBMECs were incubated with gp120 (50 ng/ml) (**a**) or METH (50 nM) (**b**) for 2, 6, and 24 h, respectively. Expression of α7 nAChR (AchR7), S100B, Tau, and RAGE, the biomarkers of Alzheimer’s-like brain pathology, was determined by Western blot using the antibodies as described in Materials and methods. 0 h: the control HBMECs without METH or gp120 stimulation. β-actin in both fractions was detected as internal loading controls.

To further examine gp120- and METH-induced expression of BBB biomarkers and receptors, HBMECs stimulated with gp120 (50 ng/ml) or METH (50 nM) at different time points were subjected to immunoblotting analysis of α7 nAChR and biomarkers [S100B, Tau, and receptor for advanced glycation end-products(RAGE)], which play an important role in Alzheimer’s-like brain pathology^13^,^16^. Our studies indicated that gp120 (Fig. 4a) and METH (Fig. 4b) were able to significantly upregulate expression of α7 nAChR, S100B, Tau, and RAGE in a time (0–24 h)-dependent manner (Fig. 4). These studies suggest that α7 nAChR, S100B, Tau, and RAGE are upregulated by gp120 and METH.

### HIV-1 gp120-, METH- and NT-induced enhancement of α7 nAChR and S100B levels *in vivo*

**Figure 5:**
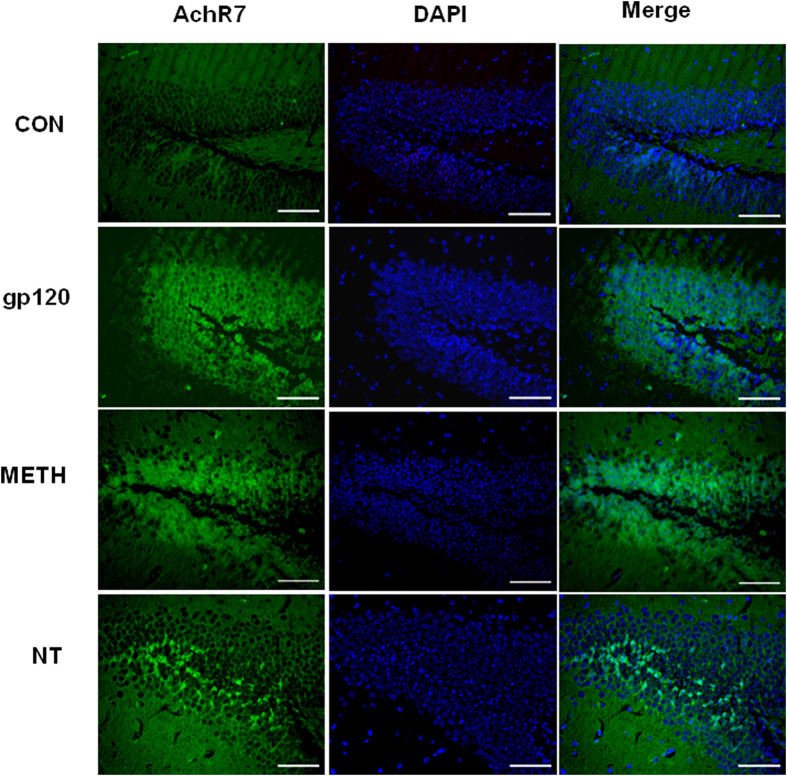
α7 nAChR expression in the dentate gyrus (DG) of the hippocampus in gp120-, METH- and NT-treated mice. Immunofluorescence microscopy showed the expression of α7 nAChR (AchR7) in DG of the hippocampus of C57BL/6J mouse brains upon treatment with HIV-1 gp120 (50 ng/mouse, 2d), METH (2, 4, 6, 8, 10, 10, 10, 10, 10, 10 mg/kg/d), and NT (1.5 mg/kg/d, 3d). DAPI was used to stain the nuclei in the brain in the merged pictures. (**CON**) mice without any treatment. **(gp120)** mice injected with gp120. **(METH)** mice injected with METH. **(NT)** mice treated with NT. Images are 200×. Scale bar = 100 μm.

**Figure 6:**
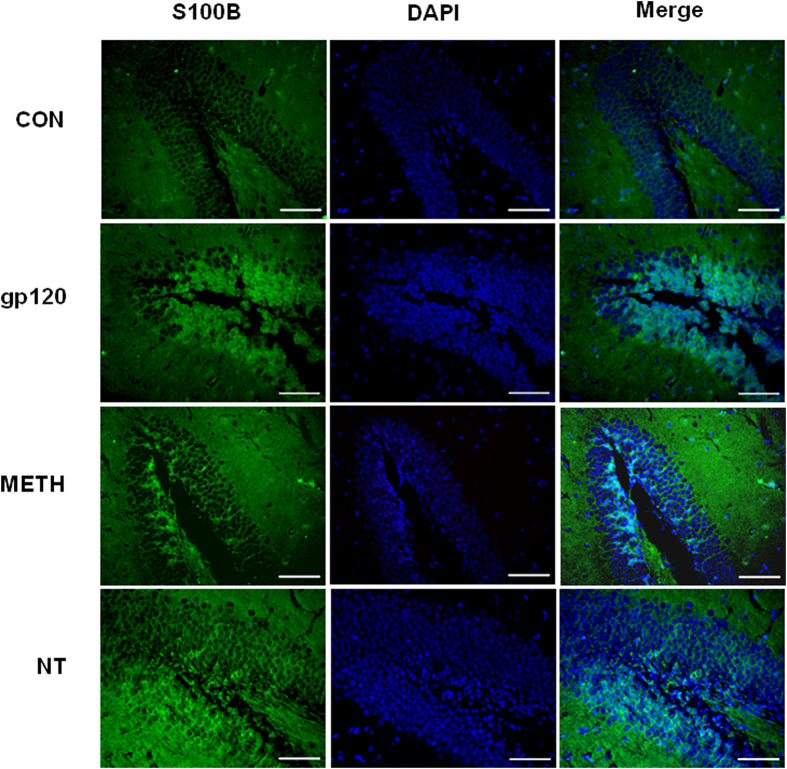
S100B expression in the dentate gyrus (DG) of the hippocampus in gp120-, METH- and NT-treated mice. Immunofluorescence microscopy showed the expression of S100B in DG of the hippocampus of C57BL/6J mice upon treatment with HIV-1 gp120 (50 ng/mouse, 2d), METH (2, 4, 6, 8, 10, 10, 10, 10, 10, 10 mg/kg/d), NT (1.5 mg/kg/d, 3d). DAPI was used to stain the nuclei in the brain tissues in the merged pictures. (**CON)** mice without any treatment. **(gp120)** mice injected with gp120. **(METH)** mice injected with METH. (**NT)** mice treated with NT. Images are 200×. Scale bar = 100 μm.

To determine whether the brain levels of α7 nAChR and S100B, which play an important role in Alzheimer’s-like brain pathology, were increased upon stimulation with HIV-1 gp120 (50 ng/mouse, 2d), METH (2, 4, 6, 8, 10, 10, 10, 10, 10, 10 mg/kg/d), NT (1.5 mg/kg/d, 3d), the expression of α7 nAChR and S100B in the hippocampal dentate gyrus (DG) in gp120-, METH- and NT-treated mice was examined by immunohistochemical staining. As shown in Figs 5 and 6, gp120, METH or NT treatment significantly increased α7 nAChR and S100B expression in the DG of the hippocampus. These findings were consistent with the *in vitro* data. It also concurred with previous studies that α7 nAChR and its agonist nicotine play an important role in the pathogenesis of CNS inflammation^10^,^11^. These results showed that gp120, METH and NT might contribute to Alzheimer’s-like brain pathology by increasing expression of α7 nAChR and S100B, the pan marker of astrocytes which are an important component of the BBB^11^,^16^. These findings suggest that α7 nAChR and S100B contribute to HIV-1 gp120-, METH- and NT-increased Aβ levels in the brain and may be involved in the increased BBB permeability by upregulating protein levels of BBB biomarkers and the cholinergic pathway.

### α7 nAChR and RAGE in HBMEC and HL60 is required for monocytes(leukocytes) transmigration across HBMECs

**Figure 7:**
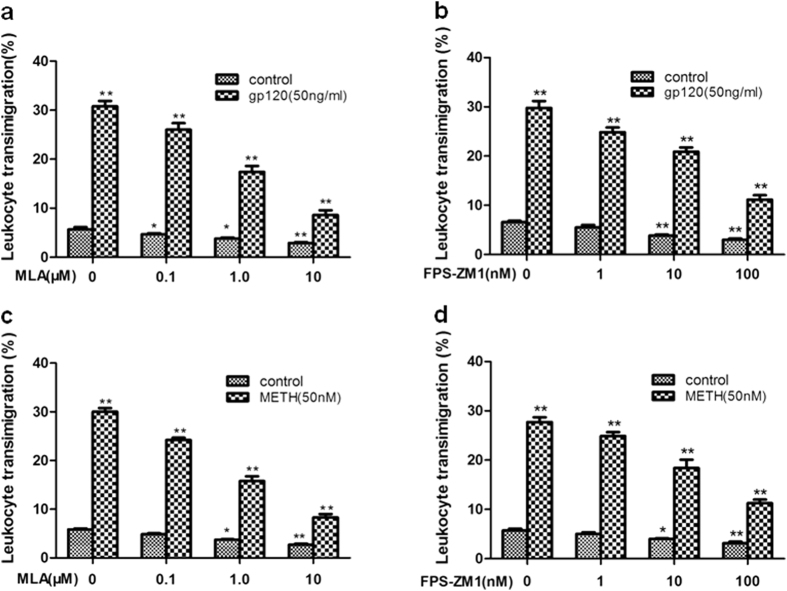
Effects of α7 antagonist (MLA) and RAGE inhibitor (FPS-ZM1) on HIV-1 gp120-, METH- and NT-induced monocyte (leukocytes) transmigration across brain endothelial cells. HBMECs were exposed to gp120 (50 ng/ml) (**a** and **b**) and METH (50 nM) (c and d) for 48 h. Effects of different doses of MLA (0–10 μM, 1 h) (**a** and **c),** FPS-ZM1 (1–100 nM, 2 h) (**b** and **d**) on monocyte transmigration with or without exposure to gp120 (50 ng/ml for 48 h) (**a** and **b**) or METH (50 nM for 48 h) (**c** and **d**). For the monocyte (leukocytes) transmigration assays, values represent the means of % transmigrating HL60 cells of triplicate samples. Bar graphs show the means ± SD of triplicate samples. *P < 0.05, **P < 0.01.

Leukocyte recruitment into the CNS plays a crucial role in the inflammatory response during Blood-brain barrier (BBB)^10^,^17^. In order to exclude the possibility that the monocyte (leukocytes) migration elicited was due to destruction of HBMECs, the integrity of the monolayer(leukocytes) was confirmed by microscopy. To examine the role of the two key regulators (α7 nAChR and RAGE) in CNS inflammation that is associated with Alzheimer’s-like brain pathology^13^,^16^, HBMECs were exposed to different doses of MLA (α7 antagonist) (0.1 to 10 μM) for 1 h and FPS-ZM1 (RAGE inhibitor) (1 to 100 nM) for 2 h prior to the HBMECs treated with (50 ng/ml gp120 or 50 nM METH for 48 h) and without gp120 or METH and subjected to monocyte (leukocytes) transmigration assays. As indicated in Fig. 7, MLA (Fig. 7a,c)) and FPS-ZM1 (Fig. 7b,d) were able to significantly inhibit leukocyte transmigration across the HBMEC monolayer treated with and without gp120 or METH in a dose-dependent manner, suggesting that α7 nAChR and RAGE expression on HBMECs and HL60 is required for gp120 or METH-enhanced monocyte (leukocytes) transmigration *in vitro*.

### α7 nAChR antagonist MLA inhibits HIV-1 gp120-, METH- and NT-induced senescence in HBMECs

**Figure 8:**
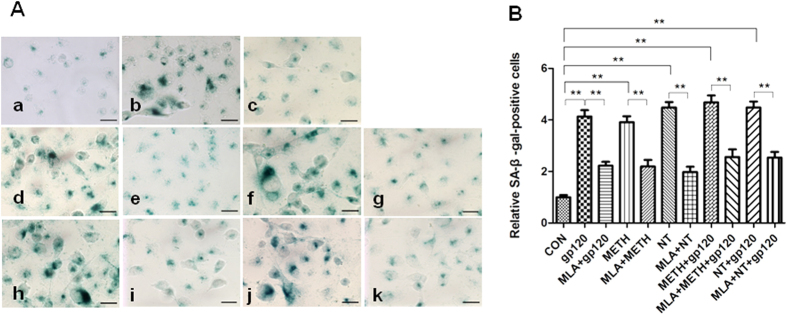
Identification of senescence cells with the SA β-gal staining assay. HBMECs were treated with MLA(10 μM) 1 h before exposure to gp120, METH, NT, METH + gp120 and NT + gp120. After 24 h exposure, the cells were stained with freshly prepared SA β-gal staining solution. Cell images were captured under a microscope at magnification 200×. The data shown are representative of three independent experiments (**A**). SA β-gal-positive values counted in three independent experiments are presented as mean values with standard deviations as described in materials and methods. The graph shows the percentage of positive cells (of the total cell number) in the treated samples (**B**). Bar graphs in (**B**) show the means ± SD of samples (**P < 0.01): (a) Control (WT HBMECs), (b) gp120 (50 ng/ml), (c) MLA + gp120 (50 ng/ml), (d) METH (50 nM), (e) MLA + METH (50 nM), (f) NT (10 μM), (g) MLA + NT (10 μM), (h) METH (50 nM) + gp120 (50 ng/ml), (i) MLA + METH (50 nM) + gp120 (50 ng/ml), (j) NT (10 μM) + gp120 (50 ng/ml), and (k) MLA + NT (10 μM) + gp120 (50 ng/ml). Scale bar = 50 μm.

To address the effect of the α7 antagonist MLA on the HIV-1 gp120-, METH- and NT-induced senescence in HBMECs, we compared the senescence of HBMECs, which exposed to gp120, METH, NT, METH + gp120 and NT + gp120 and HBMECs treated with MLA for 1 h before the treatments with these pathogenic factors. When treated with either 50 ng/ml gp120, 10 μM NT, 50 nM METH, METH (50 nM) + gp120 (50 ng/ml) or NT (10 μM) + gp120 (50 ng/ml) (24 h), HBMECs became flat and showed enlarged morphology, which is a characteristic phenotypic change in premature senescence^12^,^18^,^19^. Moreover, senescence-associated SA β-galactosidase (SA β-gal) staining was greatly increased (Fig. 8b,d,f,h,j). These morphological changes and the increase in SA β-gal stained cells confirmed that HIV-1 gp120, METH and NT efficiently induced the premature senescence of HBMECs.

However, neither the control group nor MLA treatment cells showed these morphological changes, and most of the cells maintained the normal morphology following the MLA treatment (Fig. 8a,c,e,g,i,k). Furthermor, the numbers of SA β-gal stained cells were significantly lower in both the control group with treatment and MLA-treated cells exposed to HIV-1 gp120, METH and NT (Fig. 8B). These results suggest that the **α7** antagonist MLA suppresses HIV-1 gp120-, METH- and NT-induced senescence in HBMECs.

### Cerebrospinal fluid (CSF) levels of biomarkers for Alzheimer’s-like brain pathology and BBB injury are reduced by chemical blockage of α7 nAChR (MLA) upon exposure to HIV-1 gp120, METH and NT

**Figure 9:**
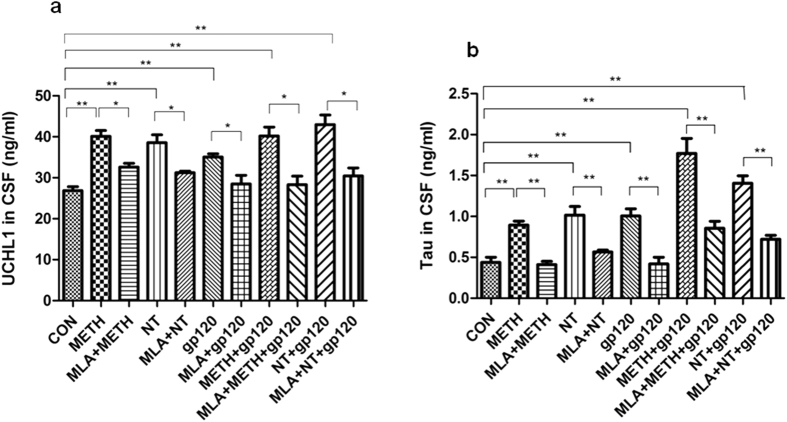
CSF Biomarker changes in neuronal injury (UCHL1) and neurodegenerative disorder (Tau). The α7 antagonist MLA could decrease CSF biomarker levels in mice upon exposure to HIV-1 gp120, METH and NT. Levels of UCHL1 (**a**) and Tau (**b**) in mouse CSF after treatment of gp120, METH, NT and MLA were examined by the ELISA assays as described in Methods and Materials. The values were expressed as concentrations of biomarkers (ng/ml) representing means of 4 samples for each group. WT mice treated with vehicle served as the control (*P < 0.05; **P < 0.01).

**Figure 10:**
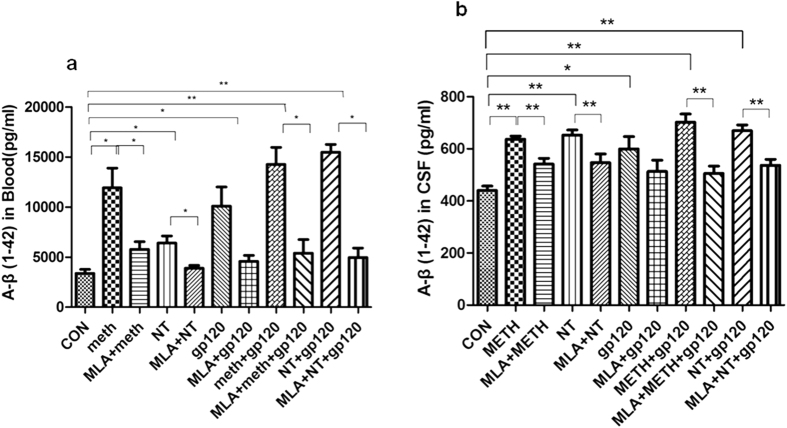
Reduction of Aβ (1–42) concentrations in blood (**a**) and CSF (**b**) samples of HIV-1 gp120-, METH- and NT-treated mice by MLA (α7 antagonist). Levels of Aβ (1–42) in blood (**a**) and CSF (**b**) of the animals after treatment of gp120, METH, NT and MLA were examined by the ELISA assay as described in Methods and Materials. The values were expressed as concentrations of Aβ (1–42) (pg/ml) representing means of 4 samples for each group. WT mice treated with vehicle served as the control (*P < 0.05; **P < 0.01).

To further determine the role of α7 nAChR in the CNS inflammation and Alzheimer’s-like brain pathology, the levels of biomarkers in CSF, including Tau, Aβ (Alzheimer’s-like brain pathology) and UCHL1 (BBB injury), were analyzed (Figs 9 and 10). The data showed that treatment with these factors alone or their combination (gp120, METH, NT, METH + gp120 and NT + gp120) could significantly increase the levels of UCHL1 (Fig. 9a)/Tau (Fig. 9b) in CSF and the concentrations of Aβ in both blood (Fig. 10a) and CSF (Fig. 10b), while the treatment with MLA resulted in a significant decrease of these biomarkers. METH/NT had a synergistic effect in increasing the Tau protein in CSF. Gp120-, METH- and NT-enhanced expression of these biomakers was significantly blocked in the animals treated with the α7 antagonist MLA. These data suggested that α7 nAChR could upregulate biomarkers of neurodegeneration, Alzheimer’s-like brain pathology and BBB injury upon exposure to HIV-1 gp120, METH and NT.

## Disscussion

Up to now, the mechanisms responsible for the pathogenesis of the CNS disorders caused by HIV-1 and drug abuse remain largely unknown. An important connection between the nervous system and the inflammatory response to diseases has been uncovered through the identification of α7 nAChR as an essential regulator of inflammation^20^,^21^,^22^. In this report, we present the evidence that HIV-1 gp120, METH and NT significantly increased Aβ accumulation in HBMECS in an α7 nAChR dependent manner. Our results also illustrated the effects of α7 nAChR on the inflammatory response and BBB integrity after treatment with HIV-1 gp120, METH and NT through upregulation of the cholinergic α7 nAChR pathway. Hence, α7 nAChR plays an essential role in Aβ homeostasis and inflammatory response in the brain, may be a shared molecular event of the neuronal response to injury incited by virotoxins that are thought to contribute to the development of cognitive deficits associated with chronic HIV-1 infection *in vivo*.

Aβ has been considered the main pathogenetic factor of Alzheimer’s disease^23^. Non-aggregated Aβ monomers have a role in the support of neuronal repair, but their conversion into oligomers with anti-parallel β-sheet structure leads to neurotoxicity^24^,^25^.

HAND encompasses the entire spectrum of neurological disorders associated with HIV-1 infection. It is proved that Nef exosomes from patients with HAND have the ability to interact with the neuroblastoma cells, subsequently increased the expression and secretion of Aβ, and finally aggravated the cognitive impairment^26^. Interestingly, patients infected with the subtype C of HIV-1 virus have a decreased incidence of cognitive deficits, confirming that Aβ peptides is linked to the HIV protein mediated neurotoxicity mechanisms^27^. The current study also demonstrated that HIV-1 gp120, METH and NT significantly increased the Aβ 1–42 levels in HBMECs, and also enhanced the Aβ accumulation in the mouse brain tissues, while MLA incubation remarkably inhibited gp120-mediated stimulation of Aβ transport. The results suggest that α7 nAChR plays an important role in Aβ homeostasis and that misregulation of the Aβ production might worsen neuronal homeostasis^28^.

Alterations of BMEC are commonly associated with HIV-1 infection. It is well-known that the integrality of the BBB plays a pivotal role in preventing microorganism invasion, and also be involved in the regulation of Aβ levels in the brain^29^,^30^. However, the mechanism of the shedding and senescence of BMECs is still unclear up to now. Previously, we have illustrated that cBMECs were used as a cellular index of the BBB damage caused by microbial (*E. coli* K1 and gp120) and non-microbial (nicotine and METH) pathogenic insults^11^. Leakage of peripheral proteins into the CNS has been used to evaluate BBB permeability^31^,^32^, such as fibrinogen or albumin. There are a number of peripheral blood biomarkers for detection of BBB injury, including UCHL1, RAGE and its ligand S100B. UCHL1, which is a component of the ubiquitin proteosome system has a more specific tissue distribution than S100B and is found more exclusively in neurons^33^,^34^,^35^. Our previous data have demonstrated that elevated serum levels of UCHL1 increased in the animals treated with gp120 and nicotine, suggesting that this protein could be used as a new molecular biomarker for BBB injury caused by microbial and nonmicrobial factors^11^. Meanwhile, RAGE and its downstream effectors, and nuclear factor-k-gene binding(NF-κB) have been shown to be involved in neuronal death and astroglial conversion to the pro-inflammatory neurodegenerative phenotype^16^,^36^. As a result, we found that the expression of Aβ, Tau and UCHL1 in CSF increased greatly after treatment with gp120, METH and NT, while had little effects on the levels of these biomarkers in the animals treated with the α7 antagonist MLA. Additionally, our data showed that gp120 or METH enhanced the proteins of α7 nAChR, S100B, Tau, and RAGE, and meanwhile efficiently induced the premature senescence of HBMECs. RAGE activation promotes vascular dysfunction by impairing endothelial nitric oxide bioavailability^37^, increasing the release of proinflammatory cytokines, thus sustaining a harmful vicious cycle enhancing vascular inflammation and BBB impairment. In this report, we have established that α7 nAChR plays an essential role in regulation of the BBB function *in vitro*. The pathogenic insult-induced HBMECs senescence, which is correlated with increased BBB permeability, is significantly reduced in the cells treated with the α7 antagonist MLA, suggesting that up-regulation of α7 nAChR is detrimental to the brain endothelial functions, and might increase Aβ transport across to the brain.

Currently, the precise mechanism responsible for the pathogenic insult mediated increase in the BBB permeability during HAND still remains elusive. Recent studies have revealed that α7 nAChR is a critical link between inflammation and neurodegeneration, which is closely associated with Alzheimer’s disease, might be a potential therapeutic target for the pathogenicities-caused by HIV-1 and drugs of abuse^38^. It has been shown that gp120 could up-regulate α7 nAChR, which is a previously unrecognized contributor to the neurotoxicity associated with HAND^38^. It has been shown that enhancing peptides (EPs) derived from the co-receptor binding region of gp120 could promptly accelerated the formation of amyloid fibrils through the EP-derived nanofibers and significantly enhance HIV-1 infection^39^,^40^. Recently, Zhang *et al* demonstrated that α7 nAChR plays a detrimental role in the host defense against CNS inflammation caused by microbial (e.g., meningitic pathogens and gp41) and non-microbial factors (e.g., METH) via the NF-κB signaling pathway^12^, which may be involved in regulation of the molecular marker (S100B) during various CNS disorders^41^. Similarly, the current study also showed that nicotine could upregulateα7 nAChR through activation of RAGE and S100B in the dentate gyrus (DG) of the hippocampus upon exposure to gp120, METH or NT *in vivo*, meanwhile the decrease of α7 nAChR and RAGE alleviated leukocyte (HL60 cell) transmigration across the HBMEC monolayers. These findings suggest that α7 nAChR is required for the modulation of inflammatory response through the cholinergic pathway, which may be involved in the pathogenesis of CNS comorbidities caused by HIV-1 virotoxins (e.g., gp120) and related factors (e.g., nicotine and METH). There are two α7 isoforms, α7 nAChR and dupα7 nAChR, that are present in human brain and innate immune system. Dupα7 nAChR is a partially duplicated α7 nAChR genetic variant^42^. It remains elusive whether and how Dupα7 nAChR may contribute to the pathogenesis of HAND in a manner different from α7 nAChR^42^. Therefore, the mechanisms underlying these pathogenicities are not well-defined until now. It is likely that a common set of genes and pathways regulated by the common receptor α7 nAChR for HIV-1 gp120, METH and NT, leading to the balanced regulation of the proinflammatory and anti-inflammatory factors in a manner dependent on the cholinergic signaling pathway.

Taken together, the major finding of the present report is that chemical blockages of α7 nAChR could significantly reduce HIV-1 gp120-, METH- and NT-induced BBB injury and CNS disorders by decreasing Aβ transport, leukocyte recruitment, cholinergic signaling, premature senescence of HBMECs and neuronal inflammation. Further insight into how HIV-1, METH and NT utilize the host cholinergic α7 nAChR pathway to augment their virulence capacity will advance our understanding of the pathogenesis and therapeutics of CNS disorders caused by multiple comorbidities.

## Methods and Materials

### Ethics statement

This study was performed in strict accordance with the recommendations in the Guide for the Care and Use of Laboratory Animals of the National Institutes of Health. Our protocols were approved by the Institutional Animal Care and Use Committee (IACUC) of The Saban Research Institute of Children’s Hospital Los Angeles (Permit number: A3276-01). All surgery was performed under anesthesia with ketamine and lidocaine, and all efforts were made to minimize suffering.

### Antibodies and reagents

All mouse monoclonal (MMAb)/rabbit polyclonal (RPAb)/goat polyclonal (PGAb) antibodies used for immunostaining, ELISA and Western blot analyses were as follows: (A) PRAb against α7 nAChR (1:60 for immunostaining, 1:500 for Western blot, Genescript, Piscataway, NJ); (B) PRAb against S100B (1:60 for immunostaining, 1:500 for Western blot, LifeSpan BioScience); (C) Rhodamine green-labeled MMAb recognizing β-Amyloid(1–40), MMAb against β-Amyloid(1–42)(1:1000 for ELISA) and PRAb recognizing Tau (Pser199/202) (1: 1000 for ELISA, 1:500 for Western blot) from Ana Spec Inc (Fremont, CA); (D) MMAb against Aβ (17–24) (1:1000 for Western blot, Covance, Emeryville, CA); (E) PRAb against UCHL1(1: 1000 for ELISA, Protein Tech); and (F) PGAbs against RAGE (sc-8230)) (1:500 for Western blot) and PRAb against β-actin (sc-7210) (1:2000 for Western blot) from Santa Cruz Biotechnology (CA). Nicotine tartrate (NT), methamphetamine (METH) and methyllycaconitine (MLA) were purchased from Sigma-Aldrich (St. Louis, MO). RAGE inhibitor (FPS-ZM1) was obtained from Calbiochem and mounting medium with 4′,6-diamidino-2-phenylindole (DAPI) was purchased from Vector (Buringame, CA). Gp120 was purchased from Immunodiagnostics (Bedford, MA).

### Brain endothelial cells and *in vitro* BBB model

HBMECs were isolated and cultured as described previously^17^,^43^. HBMECs were routinely cultured in RPMI 1640 medium (Mediatech, Herndon, VA) containing 10% heat inactivated fetal bovine serum, 10% Nu-serum, 2 mM glutamine, 1 mM sodium pyruvate, essential amino acids, vitamins, penicillin G (50 mg/ml) and streptomycin (100 mg/ml) at 37 °C in 5% CO_2_. All the phenotypes of HBMECs were confirmed as described previously^43^. HBMECs were used an *in vitro* BBB model to study Alzheimer’s-like pathology, brain endothelial permeability and senescence. Cells were treated with HIV-1 virotoxin gp120, METH and NT.

### Animal Model and Treatment Protocol

All animal experiments were performed using C57BL/6 J mice. The mice (6 week-old) were divided into 6 groups (I: Control treated with PBS; II: NT; III: METH; IV: gp120; V: METH + gp120; VI: NT + gp120)(n = 4). Two groups (II and III) of animals were exposed to low dose (1.5 mg/kg/day) of NT (oral delivery) for 3 days (twice per day) or gradually increased doses (2, 4, 6, 8, 10, 10, 10, 10, 10, 10 mg/kg from day 1 to day 10) of METH [intraperitoneal (i.p.) injection] for 10 days as described previously^10^,^11^. The animals in Group IV received daily injections from tail veins (50 ng/mouse) of endotoxin-free recombinant HIV-1 gp120 for 2 days as described previously^11^. The animals (V and VI) were exposed to METH or NT as described in groups (II and III), after METH or NT exposure, all mice received gp120 as described in group IV from Day 9 to Day 10. After perfusion through cardiac puncture with 20 ml PBS, the skull was opened. Brain tissues and CSF samples were collected as described previously^10^,^11^. The brain tissues were used to determine Aβ (17–24) protein expression by Western blot. CSF Biomarker changes in neuronal injury (UCHL1) and neurodegenerative disorder (Tau) were determined by ELISA using antibodies and antigens from Protein Tech (Chicago, IL) (UCHL1) and Ana Spec Inc (Fremont, CA) (Tau protein).

### ELISA assay

Enzyme-linked immunosorbent assay (ELISA) for Aβ (1–42), UCHL1 and Tau protein were carried out using the HBMEC culture supernatants that are the media in which the cells were growing or CSF biomarkers from C57BL/6 J mice exposed to gp120 (50 ng/ml)^11^,^44^, METH (50 nM)^11^,^44^, NT (10 μM)^10^,^11^, METH (50 nM) + gp120 (50 ng/ml) and NT (10 μM) + gp120 (50 ng/ml), respectively. Briefly, appropriate samples plus Tris buffer were applied on a microtiter plate previously coated with monoclonal anti-Aβ (1–42) (1:1000; COVANCE), anti-UCHL1 (1: 1000; Proteintech) and anti-Tau (Pser199/202) (1: 1000; Ana Spec Inc) in carbonate buffer and blocked with 1% bovine serum albumin. After washing, a peroxidase-conjugated secondary antibody was added and incubated for 1 h. After washing, Fast OPD (0.2 ml)(peroxidase substrate solution from Sigma, Milan, Italy) was added and continued incubation in the dark for 30 min. Absorbance was measured by the standard microtiter plate reader at 450 nm. Aβ (1–42), UCHL1 and Tau protein levels in the samples were determined using standard curves of Aβ (1–42), UCHL1 and Tau protein and expressed as pg/ml or ng/ml.

### Immunoblotting analysis

To assess gp120- and METH-induced expression of α7 nAChR, S100B, Tau, and RAGE in HBMECs and Aβ (17–24) protein expression from mouse brain tissues, HBMECs cultures were exposed to a wide array of insults [gp120(50 ng/ml)^11^,^44^, METH (50 nM)^11^,^44^, NT (10 μM)^10^,^11^, METH (50 nM) + gp120 (50 ng/ml) and NT (10 μM) + gp120 (50 ng/ml)], and mouse brain tissues were obtained from the animals treated with gp120, METH and NT (see above). Total protein was extracted with SDS buffer, heated and subjected to SDS-polyacrylamide gel electrophoresis (SDS–PAGE) as described previously^45^. Total protein was transferred to nitrocellulose membranes by semi-dry blotting. After blocking with 5% milk in PBSTv (PBS containing 0.1% Tween20, Sigma) for 1 hour, the membranes were probed with antibodies against α7 nAChR, S100B, Tau, RAGE, Aβ (17–24) and β-actin for 2 h. The washed membranes were incubated with the HRP conjugated secondary antibody for 1 h and then visualized using the enhanced chemiluminescence (ECL) procedure (Roche Applied Science, Indianapolis, IN).

### Transport of Aβ (1–40) across HBMECs monolayers

Confluent HBMECs cultured on Transwell filter inserts (pore size 0.4 μm, 12-well cell culture plate) were pretreated with the α7 antagonist (MLA) (10 μM) for 1 h and then stimulated with gp120 (50 ng/ml). Rhodamine green-labeled Aβ (1–40)(1 μM) was added to the upper chamber and incubated for 20 min at 37 °C. The fluorescence signal from Aβ (1–40) was measured in the lower chamber at 505 nm (excitation) and 528 nm (emission) as the indicator of transendothelial Aβ transport.

### Immunofluorescence microscopy

Mouse brains were harvested, fixed in 10% buffered formalin for 24 h, and embedded in paraffin. Sections with 5 mm thickness were prepared, deparaffinized with xylene and then rehydrated with graded ethanol and distilled water. Heat treatment in a microwave, blockage of endogenous peroxidase activity with 3% H_2_O_2_ and incubation with 10% goat serum were carried out as described in detail previously^10^. The brain tissues were stained with FITC-conjugated antibodies against α7 nAChR (Rabbit) and S100B (rabbit)^10^. The brain tissues were then mounted with mounting medium containing DAPI (from Vector). The samples were examined under a Leica fluorescence microscope at the Congressman Dixon Cellular Imaging Core Facility, Children’s Hospital Los Angeles. All pictures were taken using the same parameters to ensure that the fluorescence strength of each treatment could be compared and calculated.

### Monocytes transmigration assays

Monocytes suspensions in experimental medium were used for leukocyte transmigration assays as described previously^17^,^46^,^47^,^48^. Briefly, the confluence of the HBMECs monolayers in transwell filters (6.0 μm pore size, 6.5 mm diameter, BD Biosciences) coated with collagen was confirmed by light microscopy before the start of the assays. To test the inhibitory effects of the α7 antagonist (MLA) and RAGE inhibitor (FPS-ZM1) on gp120-, METH-and NT-induced monocyte transmigration across HBMECs. The HBMECs monolayers were pre-incubated with different doses of MLA (0, 0.1 μM, 1 μM, 10 μM) for 1 h or FPS-ZM1 (0, 1 nM, 10 nM, 100 nM) for 2 h and stimulated with gp120 (50 ng/ml) and METH (50 nM) in the upper chambers. Then, monocyte-like vitamin D3-differentiated HL60 cells (1 × 10^6^ cells/ml) were freshly prepared, added to the upper chamber and allowed to migrate over for 4 h. MLA or FPS-ZM1 was present throughout the monocyte transmigration experiment. At the end of the incubation, migrated HL60 cells were collected from the lower chamber and counted in a blinded-fashion using a hemacytometer. Final results of monocyte transmigration were expressed as the percentage of HL60 cells across the HBMECs monolayers.

### Senescence-associated β-galactosidase activity assay

HBMECs (1.0 × 10^4^/ml) were grown in chamber slides, MLA were added 1 h before treatment with gp120 (50 ng/ml), METH (50 nM) or NT (10 μM), respectively and the control group was treated with PBS. Two days post-treatment, the cells were washed with 2 ml 1x PBS, fixed in 2 ml of 3% formaldehyde, washed, and incubated for 24 h at 37 °C with a solution containing 1 mg/ml of 5-bromo-4-chloro-3-indolyl B-D-galactoside (X-Gal), 40 mM citric acid/sodium phosphate at pH 6.0, 5 mM potassium ferrocyanide, 5 mM potassium ferricyanide, 150 mM NaCl, and 2 mM MgCl2. After washing, the cell staining was viewed with a fluorescent microscope, and photographed.

### Statistical analysis

The statistical analysis of the data from our study involved analysis of variance (ANOVA). Raw data were entered into Software GraphPad Prsim 5.0 and automatically converted to the compatible format for statistical analysis packages. ANOVA and co-variates were followed by a multiple comparison test such as the Newmann-Keuls test to determine the statistical significance between the control and treatment groups. *P* < 0.05 was considered to be significant.

## Additional Information

**How to cite this article**: Liu, L. *et al*. Alpha7 nicotinic acetylcholine receptor is required for amyloid pathology in brain endothelial cells induced by Glycoprotein 120, methamphetamine and nicotine. *Sci. Rep.* **7**, 40467; doi: 10.1038/srep40467 (2017).

**Publisher's note:** Springer Nature remains neutral with regard to jurisdictional claims in published maps and institutional affiliations.

## Supplementary Material

Supplementary Information
