# Supplementary material for: Alpha7 nicotinic acetylcholine receptor is required for amyloid pathology in brain endothelial cells induced by Glycoprotein 120, methamphetamine and nicotine
Source: Sci Rep. 2017 Jan 11;7:40467. doi: 10.1038/srep40467 (PMC5225415; doi:10.1038/srep40467)

Alpha7 nicotinic acetylcholine receptor is required for amyloid pathology in brain endothelial cells induced by Glycoprotein 120, methamphetamine and nicotine

Liqun Liu[1, 2], Jingyi Yu[2, 3], Li Li[3,4], Bao Zhang[2, 3], Lingjuan Liu[1], Chun-Hua Wu[2], Ambrose Jong[2], Ding-An Mao[1*], Sheng-He Huang[2, 3*]

Figure 3a

Aβ

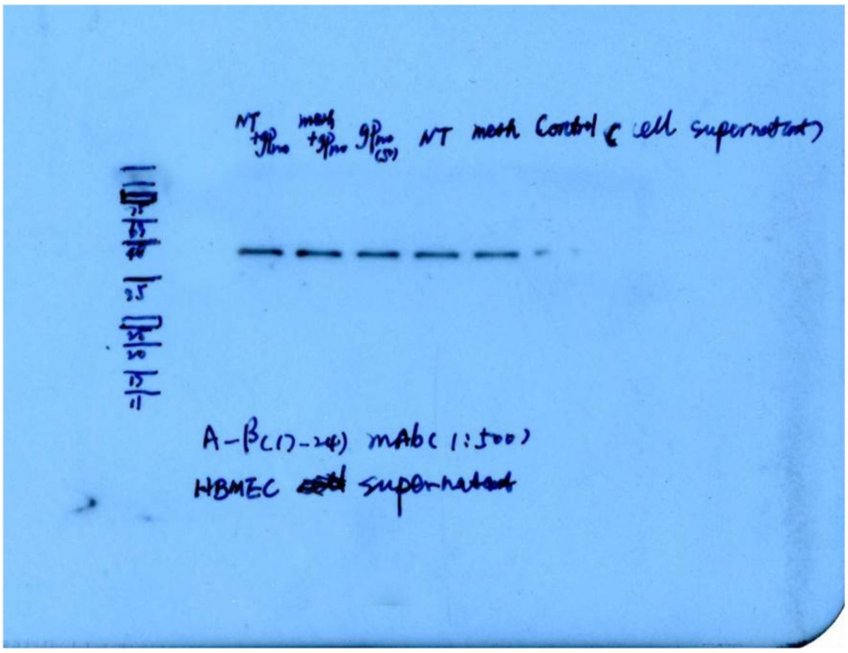

β-actin

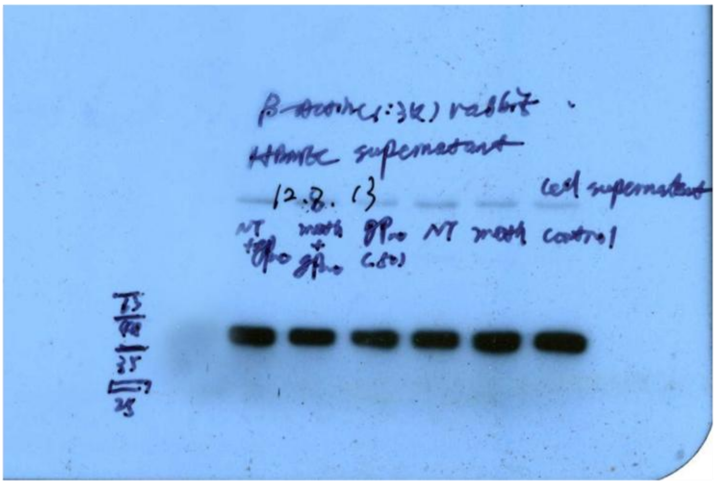

Figure 3b

Aβ

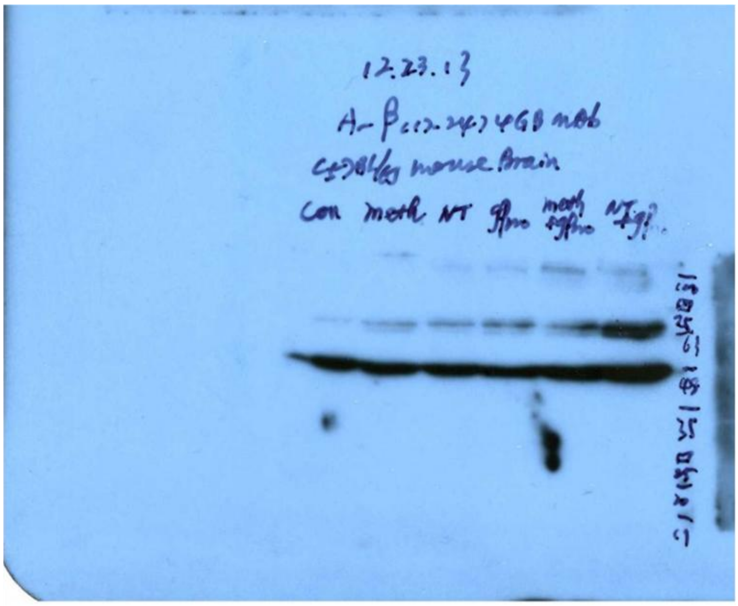

β-actin

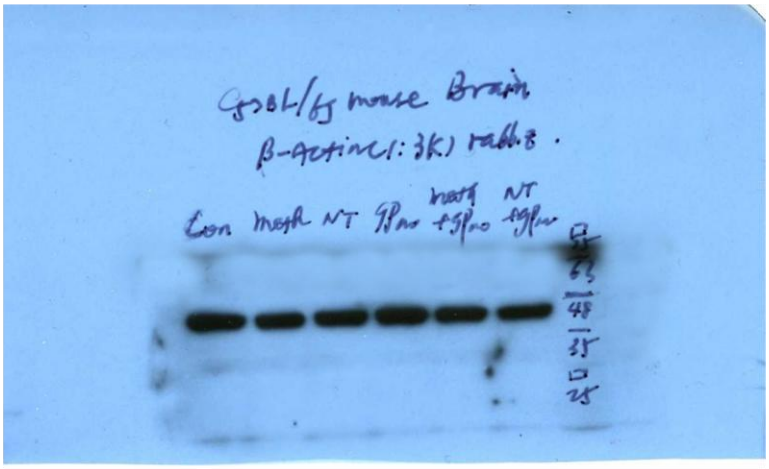

Figure 4a

AchR7, S100B, Tau, RAGE protein expression in HBMEC

after treament with gp120 (50ng/ml) （ 0h，2h，6h，24h ）

AchR7

S100B

Tau

RAGE

β-actin

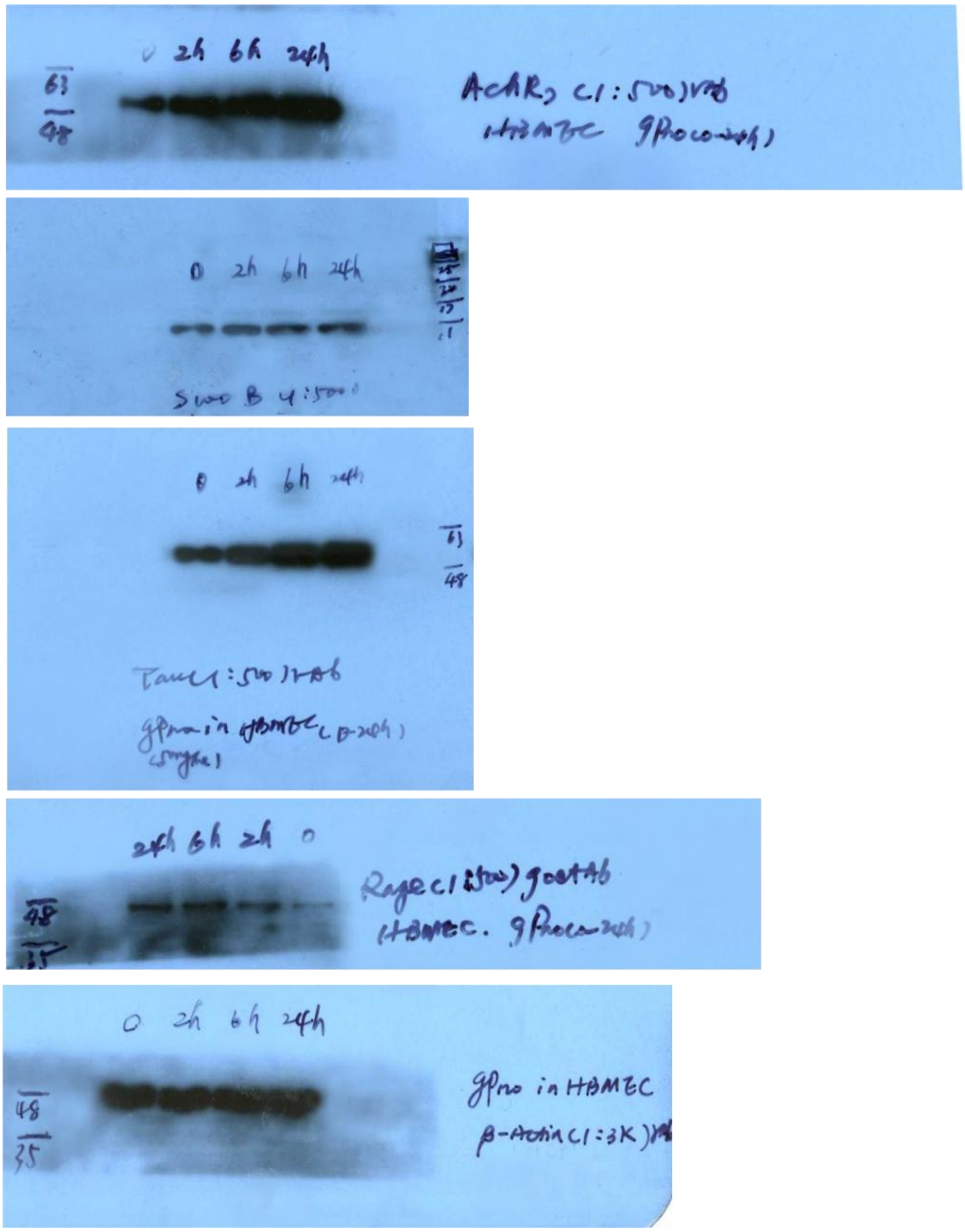

Figure 4b

AchR7, S100B, Tau, RAGE protein expression in HBMEC

after treament with METH(50nM) （ 0h，2h，6h，24h ）

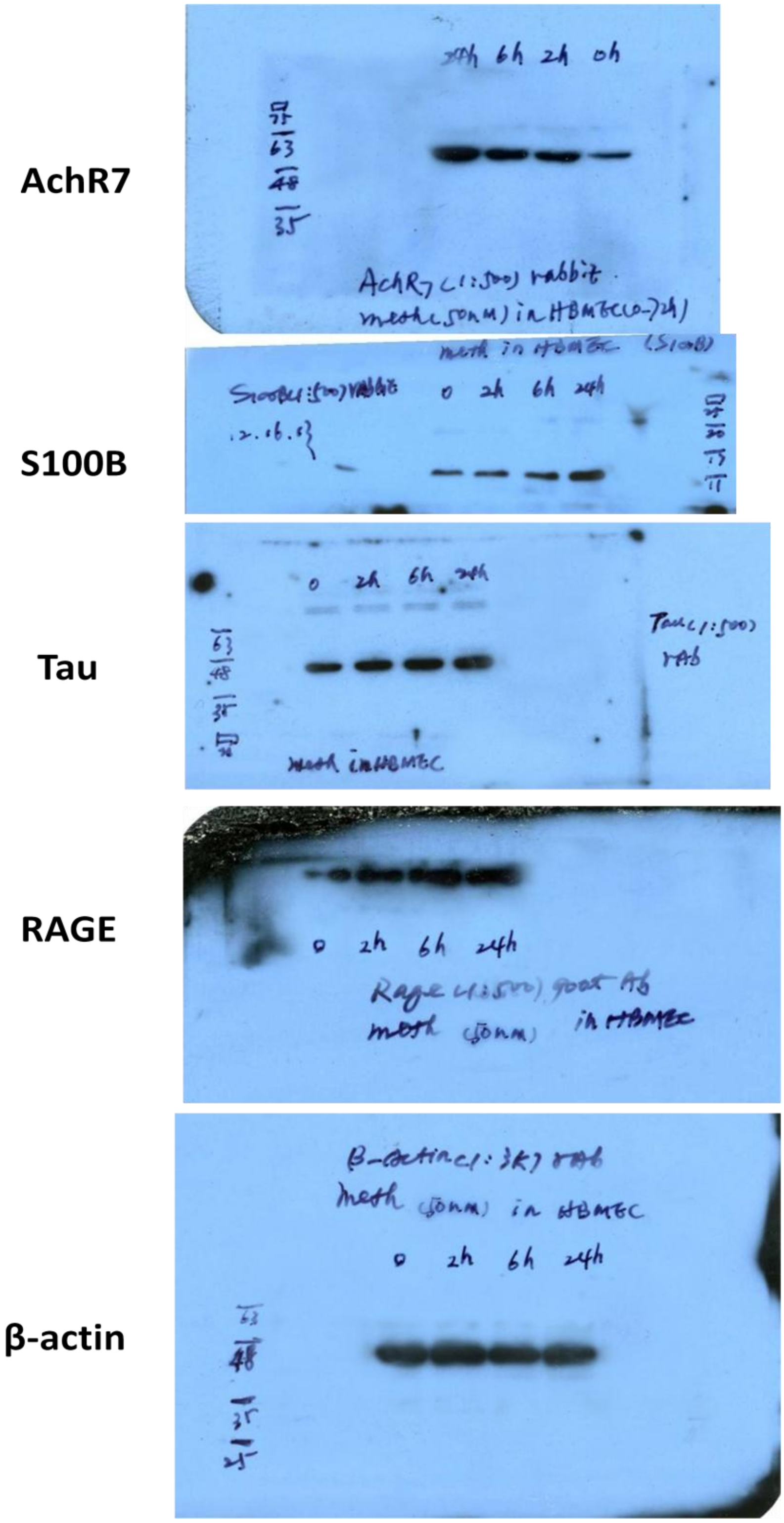

Supplement: Supplementary Information [file srep40467-s1.pdf]
